# Adequacy of oxygenation parameters in elderly patients undergoing mechanical ventilation

**DOI:** 10.1590/S1679-45082013000400011

**Published:** 2013

**Authors:** Luana Petruccio Cabral Monteiro Guedes, Fabrício Costa Delfino, Flavia Perassa de Faria, Gislane Ferreira de Melo, Gustavo de Azevedo Carvalho

**Affiliations:** 1 Universidade Católica de Brasília, Brasília, DF, Brazil.

**Keywords:** Oxigenation, Pulmonary gas exchange, Respiration, artificial, Aged, Aging, Intensive care units

## Abstract

**Objective:**

To compare ideal PaO_2_ with PaO_2_ found, ideal PaO_2_/FiO_2_ of room air with the one found, and ideal FiO_2_ with FiO_2_ found in mechanically ventilated elderly patients.

**Methods:**

Cross-sectional study that evaluated elderly mechanically ventilated patients for at least 72 hours and who underwent three subsequent blood gas analyses.

**Results:**

The sample consisted of 48 elderly with mean age of 74.77±9.36 years. There was a significant difference between the ideal PaO_2_ and the one found (p<0.001), between FiO_2_ corrected and the offered one, and also between ideal PaO_2_/FiO_2_ of room air and the PaO_2_/FiO_2_ found (p<0,001).

**Conclusion:**

A significant increase was seen in PaO_2_ and FiO_2_ and in alterations of gas exchange by PaO_2_/FiO_2_ index than those found in normal parameters.

## RESUMO

**Objetivo:**

Comparar a PaO_2_ ideal com a encontrada, a PaO_2_ ideal/FiO_2_ ambiente com a encontrada e a FiO_2_ ideal e encontrada em idosos ventilados mecanicamente.

**Métodos:**

Estudo transversal, no qual foram avaliados os idosos ventilados mecanicamente por no mínimo 72 horas e que tiveram três gasometrias seguidas.

**Resultados:**

Compuseram a amostra 48 idosos, com média de idade de 74,77±9,36 anos. Houve diferença significativa entre a PaO_2_ ideal e a encontrada (p<0,001), da FiO_2_ ofertada e a corrigida (p<0,001), e entre a PaO_2_ ideal/FiO_2_ ambiente e a PaO_2_/FiO_2_ encontrada (p<0,001).

**Conclusão:**

Observou-se aumento significativo da PaO_2_ e da FiO_2_, assim como alterações de troca gasosa pelo índice PaO_2_/FiO_2_, quando comparados com parâmetros de normalidade.

## INTRODUCTION

According to Brazilian Ministry of Health, ageing population in Brazil is growing and currently achieved more than 19 million of individuals^(1)^. Ageing brings with it an increase in chronic-degenerative diseases rate and in some cases leads to the need of interventions that require admission to Intensive Care Units (ICU)^(2)^. In 2008, pneumonia was the second leading cause of admissions among ageing population; the hypertension was the first leading cause^(3)^. Pneumonia could lead to respiratory insufficiency, which requires invasive ventilation support^(4)^. Cohen et al.^(5)^ reported that elderlies were benefited by treatment in ICU and by mechanical ventilation (MV).

Senility causes changes in several organic systems such as the respiratory system, therefore harming its function. These harms could be higher depending on environmental issues as smoking and chronic degenerative diseases^(4,6,7)^. Alterations could be divided into structurally and functionally. Enlargement of deadspace, rigidity of chest wall, decrease of mucociliary clearance, strength and muscle mass are considered examples of structural changes whereas reduction of complacency of chest wall, the increase of pulmonary complacency, changes in pulmonary capacity and volumes (reduced vital capacity and increased residual volumes) are example of functional changes^(6,8–13)^.

After define the use of MV the constant monitoring of respiratory function is fundamental to detect complications early, to enable the prognosis analysis, to evaluate response to treatment and to reduce complications related to long duration of M V, therefore enabling the weaning from ventilator as soon as possible^(14)^. Among ventilation monitoring in patients, the following could be used: pulse oximetry, gasometry (blood gas analysis), measurement of oxygenation and ideal fraction of inspirited oxygen (FiO_2_), among others. The PaO_2_/ FiO_2_ ratio is used to determine patient's oxygenation index, which is also called pulmonary oxygenation capacity index. The PaO_2_/FiO_2_ relations >301 indicate an adequate oxygenation between 201 and 300, acute lung injury (ALI); and relations <200 characterize a sign of acute respiratory distress syndrome (ARDS)^(15–17)^. In ICUs the measurement of pulmonary oxygenation capacity index is important in order to monitor each patient in the most accurate way.

Age is included in the calculation of ideal PaO_2._ The measurement is carried out with patient in supine position, and it uses the following formula: PaO_2_= 109-(0.43 x age)^(18)^. For this reason, elderly people commonly present a lower PaO_2_. Mechanical ventilated patients receive higher amount of FiO_2_ than room air (21%), which is called oxygenotherapy. The FiO_2_ delivered could be corrected using the corrected FiO_2_ formula=(delivered FiO_2_ x ideal PaO_2_/PaO_2_ found)^(8)^. When oxygen (O_2_) is administered in high doses and for longer periods, it might generates deleterious effects such as absorption atelectasis, interstitial pulmonary edema, and changes in cell structure and function^(19,20)^. The use of high doses of O_2_, besides being potentially harmful for patients, increases costs in hospital stay because of O_2_ supplement high cost.

## OBJECTIVE

To analyze parameters of gas exchange expected and found in mechanically ventilated elderly patients considering ideal and found PaO_2_, ideal and found PaO_2_/FiO_2_ index and corrected and delivered FiO_2_. We hypothesize that mechanically ventilated elderly patients are receiving FiO_2_ beyond necessary, and as a consequence they are subject to deleterious effects of high doses of O_2_. Once a different relation is established, which is corrected by age, concerning the PaO_2_/FiO_2_ relationship, patients would receive lower doses of FiO_2_. In addition, this establishment would enable few numbers of possible ALI/ARDS cases and complications due to O_2_ toxicity.

## METHODS

This was a quantitative, cross-sectional and descriptive study. Data were collected from patients' medical records admitted at the hospital where the study was conducted from January 1^st^ to May 31^st^ 2011.

Inclusion criteria were men and women aged 60 years or older admitted at the ICU independently of the diagnosis, and submmitted to MV for at least 72 hours and blood gas analysis during three consecutive days after MV.

We excluded those patients who died before 72 hours after invasive MV, not underwent blood gas analysis within the first 3 days after invasive MV and evolved with airway extubation before 72 hours of MV.

Patients' medical records were used to collect information. We instructed an assistant researcher graduated in physiotherapy to use a form created by the researchers of this study; this assistant was the responsible to collect the data. In order to create an epidemiological profile of patients, information were collected using a nursing notepad that included patients' name, age, gender and diagnosis. Medical records and the nursing notepad were analyzed in the ICU, and no documents were taken outside the unit.

During patients' hospitalization, in the morning, a nurse collected the sample using a BD 3mL or 5mL syringe (Becton, Dickinson and Company, New Jersey, USA) for blood gas analysis. We did not request to change the collection time because our study analyzed only medical records. A laboratory technician or the on-duty nurse collected the blood gas analysis. After that, samples were analyzed by a laboratory hired by the hospital. The laboratory informed that the blood gas analyzer (Gem® Premier™ 3000, Instrumentation Laboratory, Hamburg, Germany) was automatically calibrated after the use. Medical records lack the information if collection was done through radial or femoral artery.

Data from 3 consecutive days since the first day that patient underwent MV were used. We observed blood gas analysis and adequate MV based on these data regarding the delivered FiO_2_, the PaO_2_ considered ideal, and also the SpO_2_.

This study was approved by the Ethical and Research Committee of the *Universidade Católica de Brasília* (UCB), protocol n. 328/2010.

A sample descriptive analysis was conducted using means, standard deviation and frequencies. We used the paired Student's t-test to compare ideal and found PaO_2_, corrected and delivered FiO_2_, and relation between ideal and found PaO_2_/FiO_2_. A of CI95% and p≤0.05 were considered for the analysis. For statistical analysis we used the Microsoft office Excel 2007 for Windows and the Statistical Package for the Social Science (SPSS) program (version 14.0; SPSS Inc., Chicago, IL, USA).

## RESULTS

A total of 335 patients were admitted to hospital ICU from January 1^st^ to May 31^st^ 2011, of them 177 were men. Patients' mean age was 62.83±18.56 years with maximum of 101 and minimum of 13 years old, being 61.50% inpatients aged 60 years or over. The mean hospital stay was 11.15±26.23 days with maximum of 222 and minimum of 1 day. In all, 82.93% of patients stayed in the ICU for 11 days. Of these inpatients 23.28% died, 5.37 were transferred and 71.34% were discharged.

Among patients admitted to ICU in the period, 50 fulfilled the study inclusion criteria. At the beginning, data normality was assessed using the Kolmogorov-Smirnov test and no deviations were seen. However, two outliers were found later, but they were excluded from the sample. Sample was composed by 48 patients with mean age of 74.77±9.36 years old with maximal of 101 and minimal of 60 years old; of them 25 were women. The mean time of hospital admission was 37.10±38.93 days with maximal of 184 days and minimal of 3 days. Of included patients 77.08% died, 20.83% were discharged and 2.08% were transferred. In moment of hospital admission 38% of patients were diagnosed with more than one disease. Main events are described in table 1.

**Table 1:** Main events of study population

Intercurrences	%
Pulmonary	32
Cancer	8
Circulatory	12
Surgeries	3
Renal	4
Neurologic	19
Metabolic	3
Gastrointestinal	7
Others	12

Among events in the study, diseases with higher incidence are described in table 2.

**Table 2:** Most frequent diseases found among higher incidence events

More frequent diseases	%
Cardiopulmonary arrest	2
Coronary disease	2
Aortic aneurysm	2
Acute myocardial infarction	2
Systemic hypertension	5
Congestive heart failure	7
Parkinson's disease	5
Alzheimer's disease	5
Reduced level of consciousness	7
Hydrocephalus	2
Stroke	11
Subdural hematoma	2
Chronic obstructive pulmonar disease	11
Pneumonia	13
Acute respiratory failure	24

There was a statistical difference (p=0.001) among ideal values of PaO_2_ (74.84±4.04) and values found (124.89±20.33), as well as between ideal PaO_2_/FiO_2_(365.92±19.21) and found PaO_2_/FiO_2_ (288.29±100.06), delivered FiO_2_ (0.49±0.15) and corrected FiO_2_ (0.32±0.12), p=0.001. Patients have had PaO_2_ 62.90% above ideal mean and FiO_2_ 54.20% above mean that should be provided. In addition, gas exchange 21.20% lower than ideal level. These comparisons could be seen in table 3.

**Table 3:** Comparisons between ideal values and those found in study population related to partial pressure of oxygen inspired oxygen fraction and relation between partial pressure of oxygen/ inspired oxygen fraction

	Ideal	Found	Difference from ideal (%)	p value
PaO_2_	74.84±4.04	124.89±20.33	60.90	0.001*
FiO_2_	0.32±0.12	0.49±0.15	54.20	0.001*
PaO_2_/FiO_2_	365.92±19.21	288.29±100.06	21.20	0.001*

* p≤0.05. Values expressed in means, standard deviations and difference in percentage.

PaO_2_: partial pressure of oxygen; FiO_2_: inspired oxygen fraction; PaO_2_/FiO_2_: partial pressure of oxygen/inspired oxygen fraction.

## DISCUSSION

Paiva et al.^(21)^ analyzed the epidemiologic profile of patients admitted to ICU during 7 years. They observed that 47.37% of patients were 60 years or older. Rocha Hernández et al.^(22)^ also reported that patients over 60 years old comprises the highest percentage of those admitted to ICU. Our results corroborate with these studies because 61.50% of patients admitted to ICU were 60 years or older.

In the present study, the most frequent disease was acute respiratory failure followed by pneumonia and stroke. In Paiva et al.^(21)^ study, most prevalent disease in patients older than 60 years were acute myocardial infarction and acute respiratory failure. This latter high prevalence emphasizes the need for further studies on the adequate FiO_2_, once acute respiratory failure evolves quite inevitable to MV and, as a consequence, to the administration of supplemental O_2_, which when poorly administered can lead to toxic effects.

Based on patients' age their PaO_2_ were proportionally higher than the ideal PaO_2_. This information is relevant because free radicals interaction have been described as an increase in “disease status” that include inflammation and ischemia^(23)^. Main consequences of such free radicals interaction are damage to cell membrane, enzyme inactivation and changes to the molecular genetic material^(24)^. Halliwell et al.^(25)^ stated that O_2_ is widely used in clinical medicine, however, this use must not blind the professional understanding concerning its toxic effects because present O_2_ in inspired air slow damage effects that depend on patients' organism, age and nutritional status. On the other hand, in a study conducted in pigs by Aoki et al.^(26)^ the deleterious effects of low doses of O_2_ (40%) were more remarkable within the first 4 weeks of administration than by 8 weeks in which the damages were stagnated. In our study, measures of FiO_2_ and PaO_2_ were conducted for a short-term, which indicates that a careful attention to rigorous administration of this gas is necessary; this regulation could be done using the ideal PaO_2_.

Metnitz et al.^(27)^ reported that patients diagnosed with ARDS have a highly compromised antioxidative system, both by administration of high FiO_2_ and inflammatory status. In the present study, although the elderlies were not diagnosed with this syndrome, the population presented high doses of FiO_2_ and, in several cases, inflammatory status due to underlying disease. Li et al.^(28)^ observed that rats exposed to high doses of FiO_2_ developed similar lung conditions to those found in patients with ARDS. Therefore, PaO_2_ and FiO_2_ control is fundamental to MV patients' recovery.

We observed that the PaO_2_/FiO_2_ relation found lead to misinterpretation of gas exchange condition because patients presented a PaO_2_ extremely above the ideal level and, as a consequence, the relation that results in this index was even lower. Lang et al.^(29)^ observed high levels of free radical markers in patients who received high FiO_2_ and who were at risk of developing an important inflammatory process and finally ARDS, which again raises the importance of a rigorous PaO_2_ control to calculate PaO_2_/FiO_2_, an adequate indicator of this high mortality syndrome especially in ageing individuals who already have important changes in lungs due to ageing.

Other issue that emphasizes the need to control FiO_2_ administrated to ageing people is the statement by Araújo Neto^(20)^ that O_2_ toxicity is age dependent placing ageing individuals at higher risk of complications.

To reduce what could be wrongly considered low PaO_2_/FiO_2_ relations, we suggest to consider the ideal PaO_2_/FiO_2_ calculation, *i.e.*, the ideal gas exchange of that particular patient will be the ideal PaO_2_ divided by room air FiO_2_. Such “ideal” values would be considered when delivered FiO_2_ and found PaO_2_ are adjusted. Therefore, it is important to emphasize that PaO_2_ range in hemoglobin dissociation curve ranged from 60 to 100mmHg for an appropriate saturation. The ideal PaO_2_ calculation for people aged until 100 years old refers to PaO_2_ within the range (66mmHg) showing that to use this formula as a parameter is adequate. Therefore, it is advisable to calculate PaO_2_ to adjust the FiO_2_.

Metnitz et al.^(27)^ showed that nutrient replacement with antioxidative capability, which helps the organism to fight against free radicals, was not enough to compensate the needs of patients submitted to increased O_2_ fraction. Del Maestro study^(23)^ affirmed that in cases of ischemia there is the need to administrate O_2_ adequately and give enough time for cellular mechanisms to protect themselves against free radicals that could adapt and neutralize actions of these aggressors. In this way, such actions could avoid damages to cells, which are not seen in clinical practice, but can be seen in our study by PaO_2_ increase.

For this reason, we believe that increased PaO_2_ is harmful both for the toxic effects caused by O_2_ and for the false impression of poor gas exchange that most of the times guide MV therapy. Our study did not include correlated increased PaO_2_ with mortality. Patients' severity was not analyzed in our study, however, this issue should be considered in future analyses.

There is the hypothesis that ageing patients could be affected by O_2_ toxicity because high doses of O_2_ were delivered.

The present study limitation was the absence of a precise measurement of time elapsed between the collection and the blood gas analysis. However, the service where the collection was done has the protocol of analyzing the material immediately upon receive in order to ensure the exam's accuracy. We did not observe clinical impacts by administration of high doses of O_2_. The scarcity of references in the literature on the subject was also a limitation because the studies found were outdated or used animal model. Considering the scarcity of researches in the field, further studies seem warranted.

## CONCLUSION

In the study population no significant differences were seen between normal parameters and found PaO_2,_ as well as alterations of exchange according to PaO_2_/FiO_2_ and FiO_2_ delivered.
